# Supplementary figures and images for: Utilizing mutual information for detecting rare and common variants associated with a categorical trait
Source: PeerJ. 2016 Jun 16;4:e2139. doi: 10.7717/peerj.2139 (PMC4918222; doi:10.7717/peerj.2139)

A

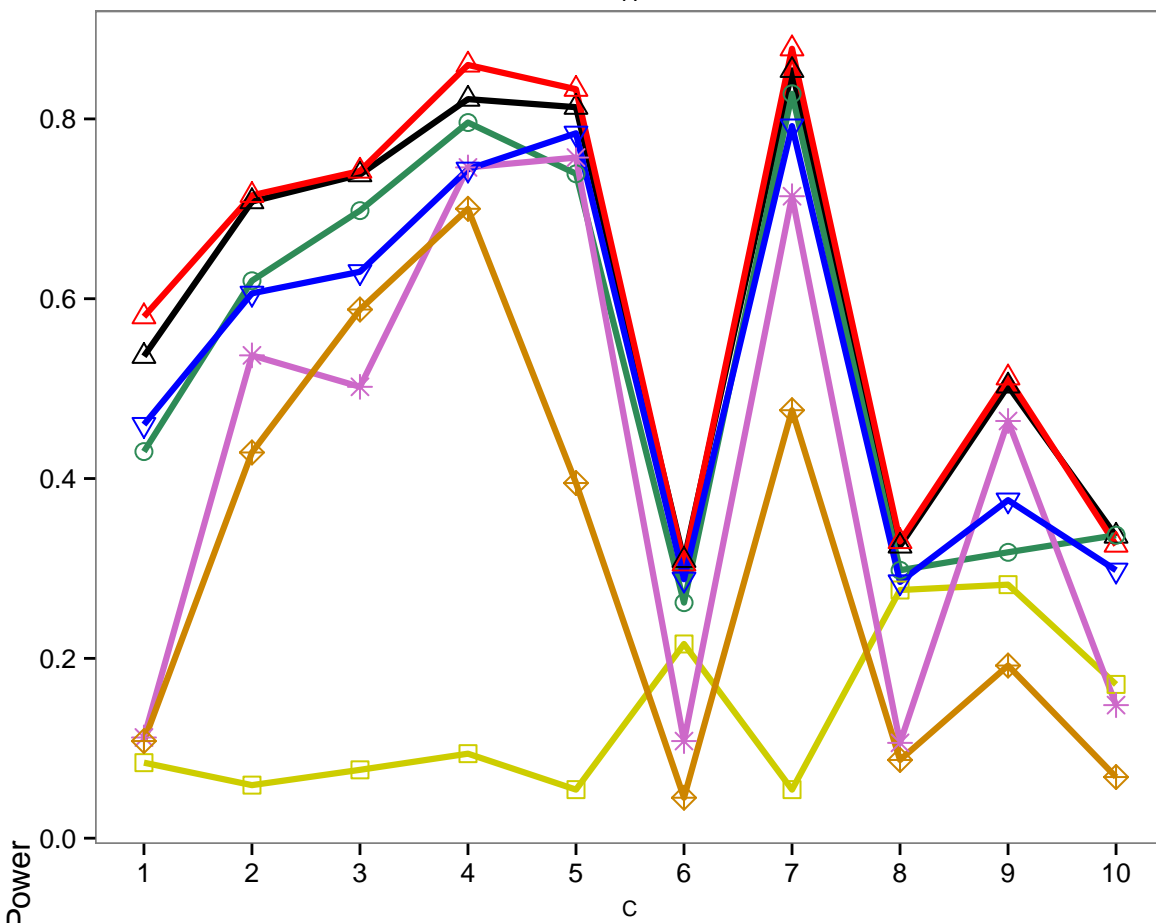

B

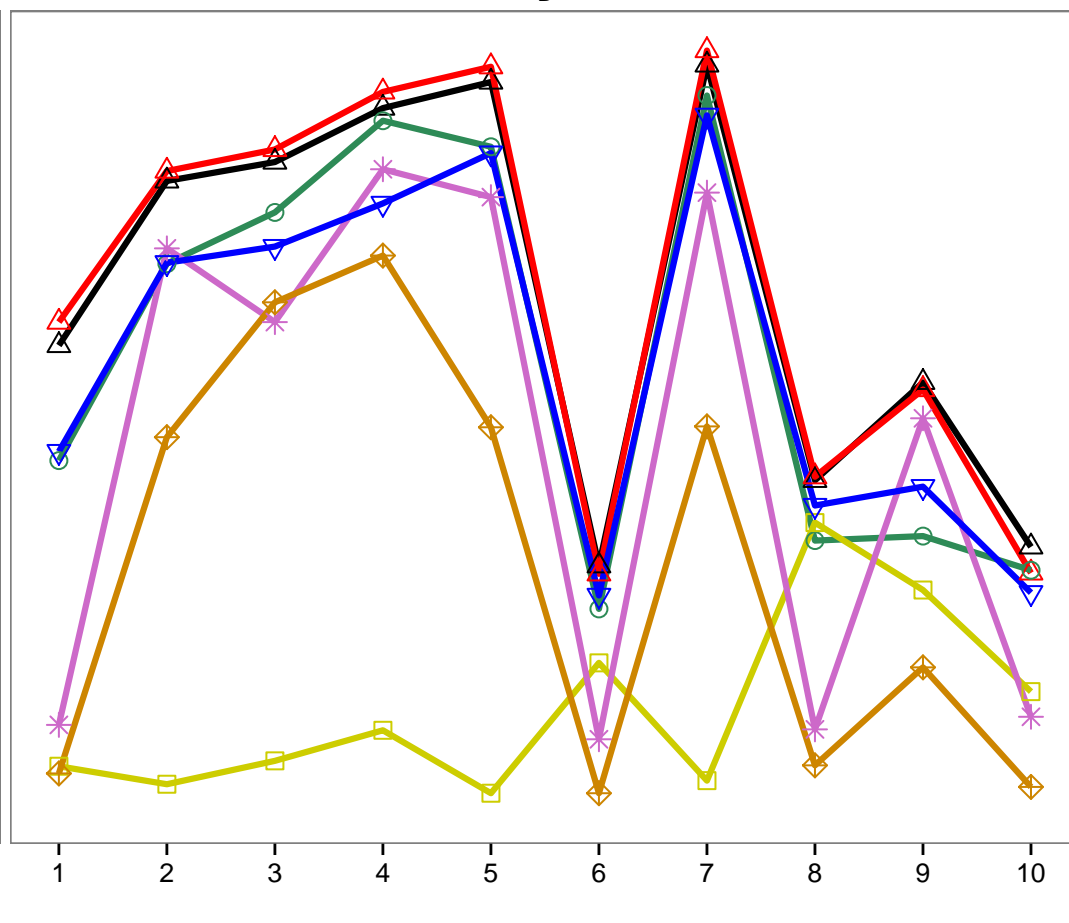

C

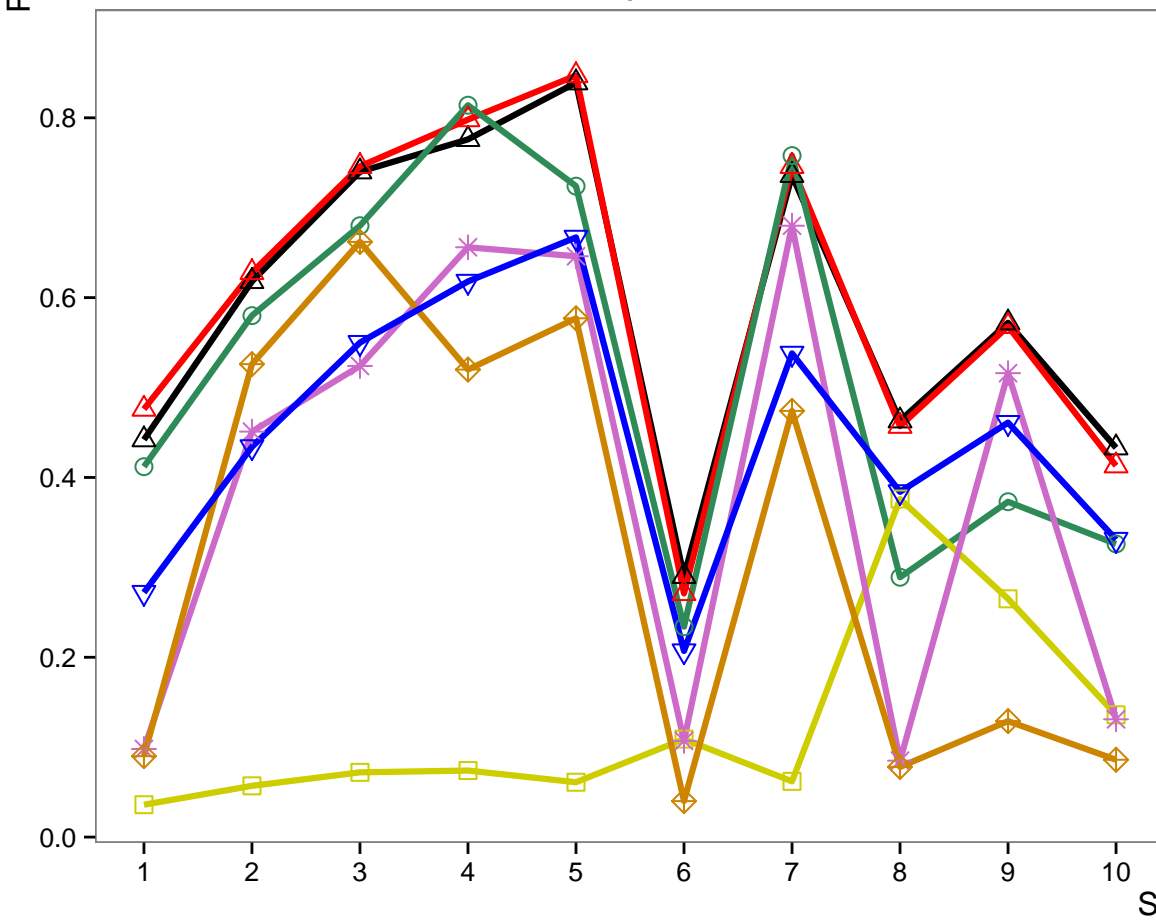

Tests

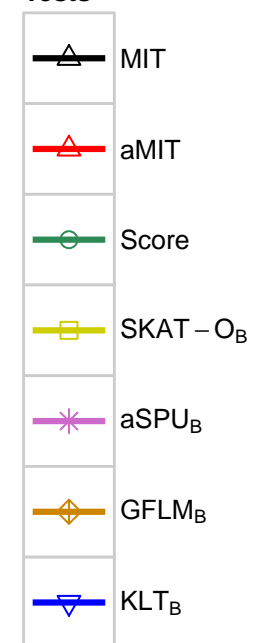

Supplement: Figure S1 — Power results of seven tests with 1,000 individuals for trait with five categories. (A) ρ = 0; (B) ρ = 0.5; (C) ρ = 0.9. [file peerj-04-2139-s003.pdf]

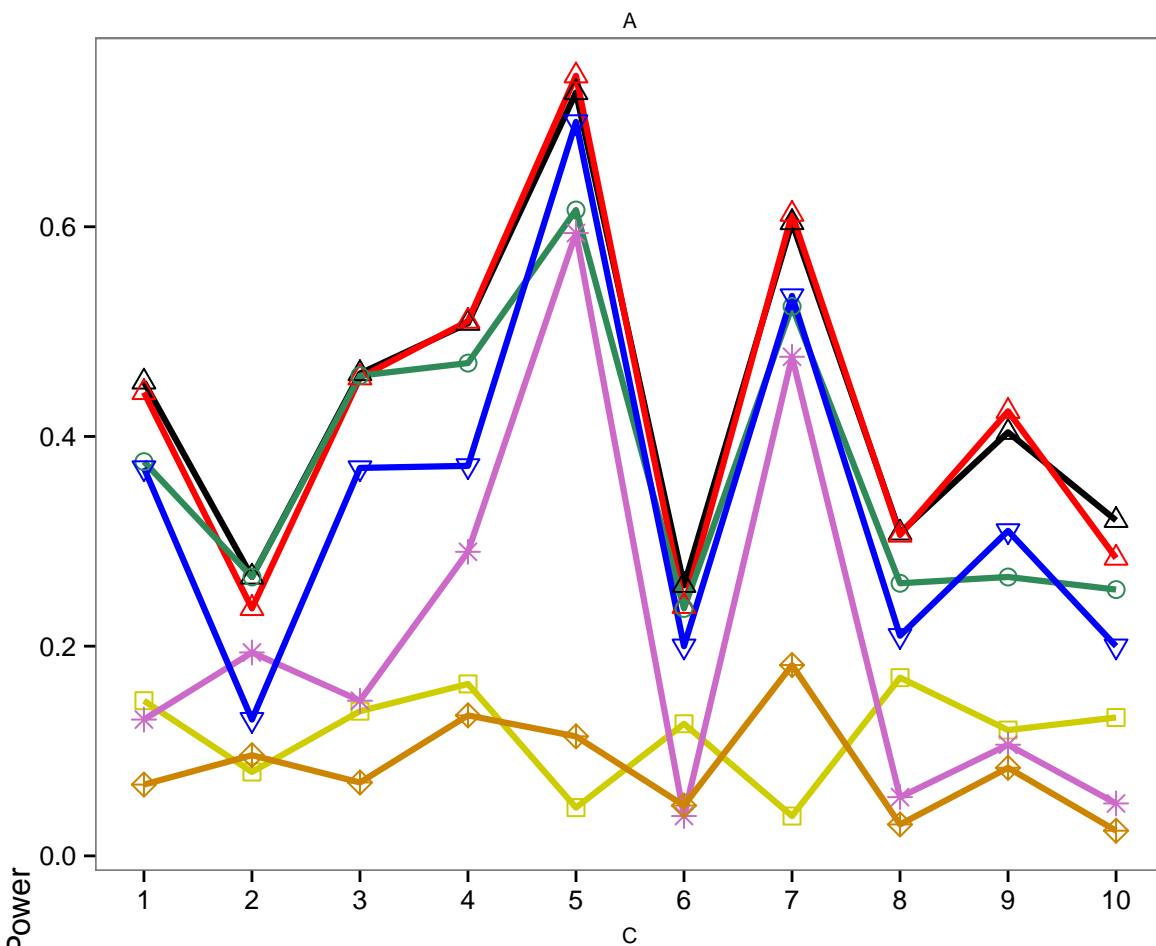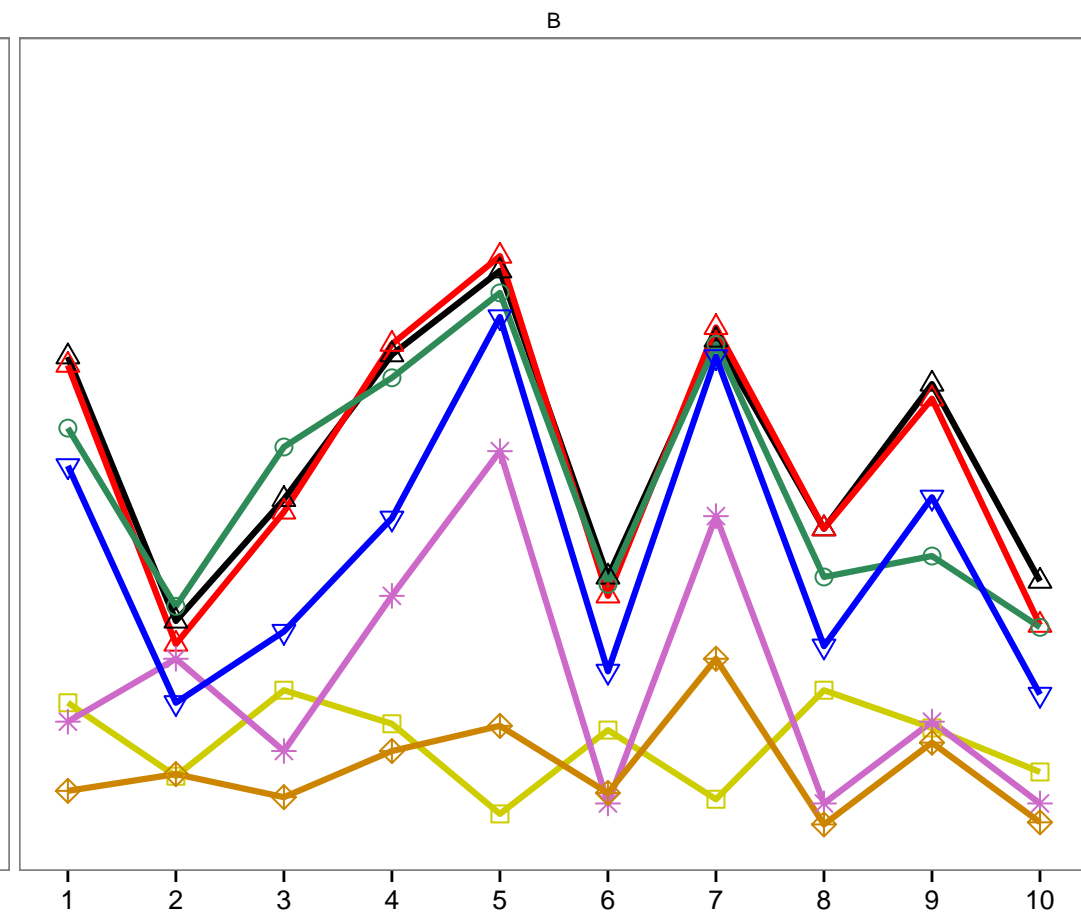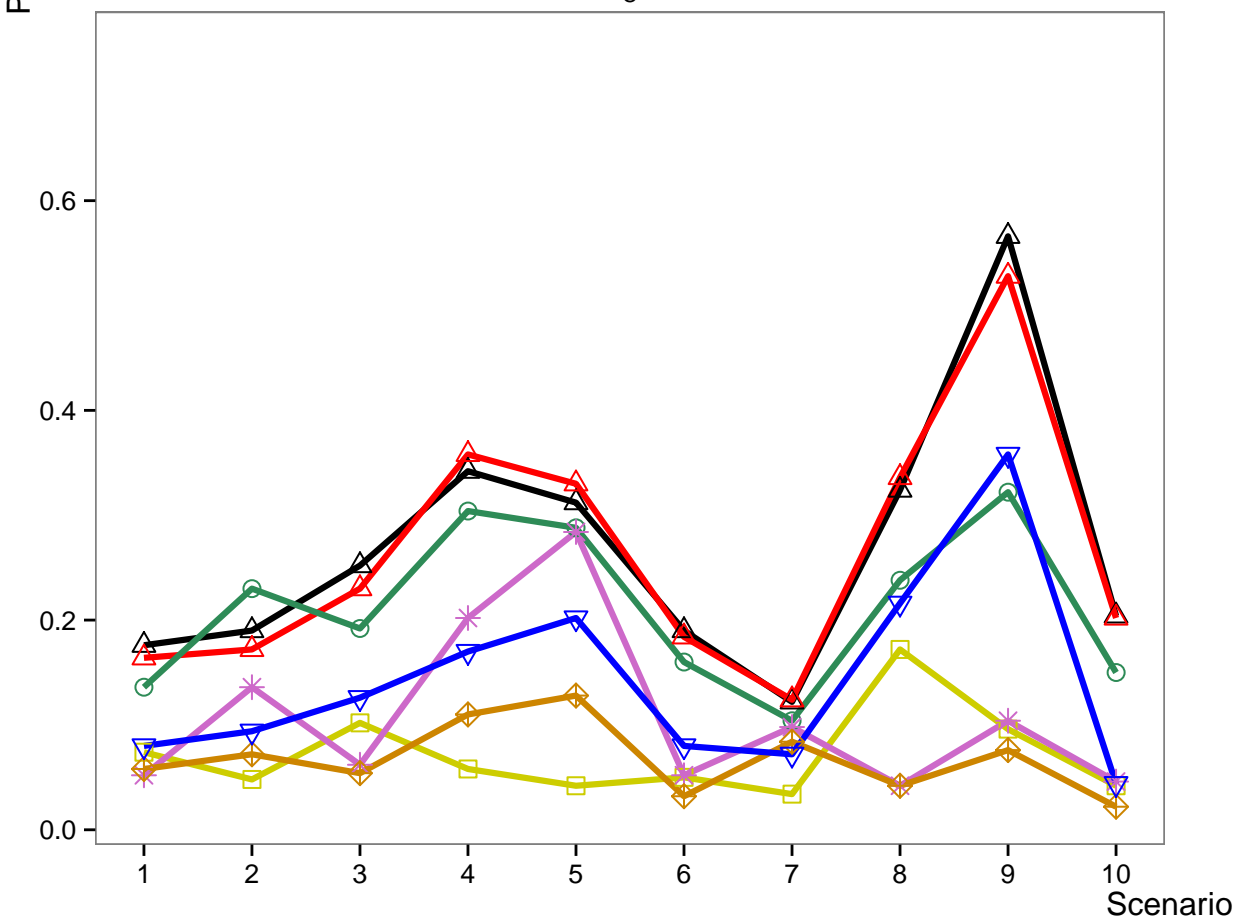

Tests

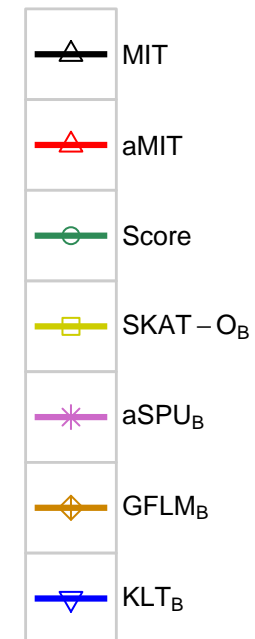

Supplement: Figure S2 — Power results of seven tests with 1,000 individuals for trait with eight categories. (A) ρ = 0; (B) ρ = 0.5; (C) ρ = 0.9. [file peerj-04-2139-s004.pdf]

## ALCOHOLISM

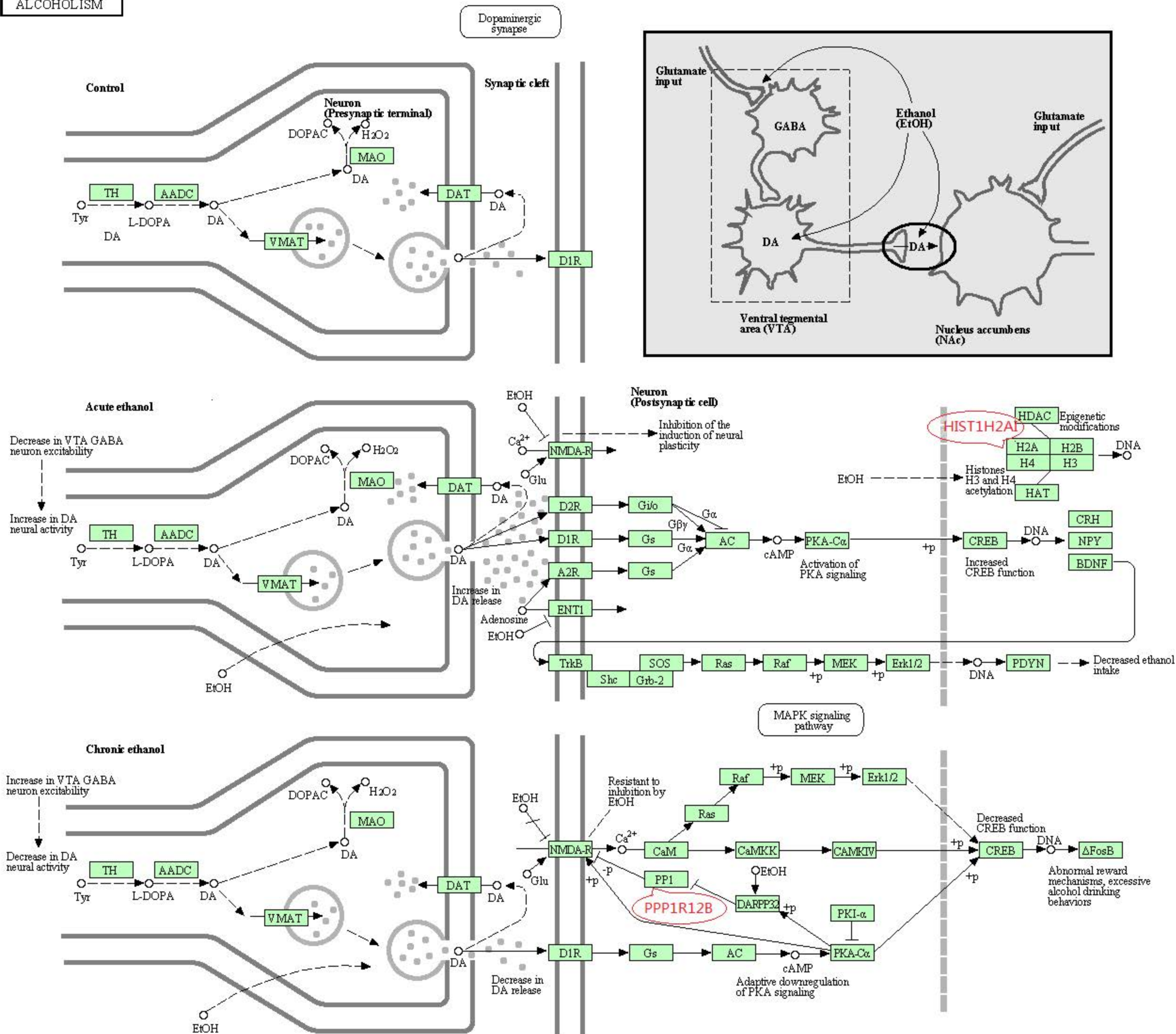

Supplement: Figure S3 — The alcoholism pathway for human from KEGG. [file peerj-04-2139-s005.pdf]
