# Supplementary material for: Utilizing mutual information for detecting rare and common variants associated with a categorical trait
Source: PeerJ. 2016 Jun 16;4:e2139. doi: 10.7717/peerj.2139 (PMC4918222; doi:10.7717/peerj.2139)
Supplement: Supplemental Information 3 [file peerj-04-2139-s008.pdf]

Package ‘MIT’

May 20, 2016

Type Package

Title Utilizing Mutual Information for Detecting Rare and Common Variants Associated with a Categorical Trait

Version 0.1.0

Author Leiming Sun, Chan Wang and Yue-Qing Hu

Maintainer Yueqing Hu <yuehu@fudan.edu.cn>

Description This package is used for the association study between genetic variants and a categorical trait. The package is for MIT and aMIT. The phenotype is a categorical trait. Each column of genotype matrix is for an SNP and the number of genetic variants is more than one.

License GPL

LazyData TRUE

R topics documented:

MIT_aMIT 1

Index **3**

`MIT_aMIT` *MIT and aMIT*

Description

Nonparametric tests MIT and aMIT based on mutual information detect association between genetic variants in a gene/region and a categorical trait. MIT and aMIT can gauge the difference among the distributions of rare and common variants across a region given every categorical trait value. The significance is determined by a permutation procedure.

Usage

```
MIT_aMIT(X,Y,B=1000)
```

Arguments

X	Genotype matrix with dimension n (#individuals) by m (>1) (#genetic variants), i.e. (i,j) entry is the # minor allele at jth variant for ith individual, i=1,...,n; j=1,...,m
Y	Vector of phenotype of length n, i.e. ith component is the categoical trait value of the ith individual, taking value from 1,2,...,K
B	Positive integer indicating the number of permutations (1000 by default)

Details

There is no missing data.

Value

The two p-values of the test statistics MIT and aMIT

Author(s)

Leiming Sun, Chan Wang and Yue-Qing Hu

References

Leiming Sun, Chan Wang and Yue-Qing Hu. Utilizing Mutual Information for Detecting Rare and Common Variants Associated with a Categorical Trait, PeerJ, 2016, in press

Examples

```
genotype<-matrix(sample(c(0,1,2),1000,replace=TRUE),500,2) ### 500 individuals and 2 SNPs
phenotype<-c(rep(1,100),rep(2,200),rep(3,200)) ### a categorical trait and K=3
pvalue<-MIT_aMIT(genotype,phenotype,B=1000)
pvalue_MIT<-pvalue[[1]]
pvalue_aMIT<-pvalue[[2]]
```

Index

∗Topic **MIT and aMIT**
MIT_aMIT, 1

MIT_aMIT, 1
