# Supplementary material for: Utilizing mutual information for detecting rare and common variants associated with a categorical trait
Source: PeerJ. 2016 Jun 16;4:e2139. doi: 10.7717/peerj.2139 (PMC4918222; doi:10.7717/peerj.2139)
Supplement: Supplemental Information 4 [file peerj-04-2139-s009.zip › MIT/html/00Index.html]

R: Utilizing Mutual Information for Detecting Rare and Common
Variants Associated with a Categorical Trait

# Utilizing Mutual Information for Detecting Rare and Common Variants Associated with a Categorical Trait

---

## Documentation for package ‘MIT’ version 0.1.0

- DESCRIPTION file.

## Help Pages

|  |  |
| --- | --- |
| MIT\_aMIT | MIT and aMIT |
